# The Effect of Homecare Team Visits in Terminal Cancer Patients: Role of Health Teams Reaching Patients Homes

**DOI:** 10.4103/0973-1075.58463

**Published:** 2009

**Authors:** Pratik Banerjee

**Affiliations:** Cansupport, Near Kanak Basti, RK Puram, Delhi, India

**Keywords:** Community network officers, Counselor, Doctors, Homecare teams, Nurses

## Abstract

**Aim:**

The study has been conducted to see the effectiveness of homecare teams visit in terminal cancer patients (palliative care).

**Materials and Methods:**

The study basically utilized the effectiveness of the Cansupport's functioning. Cansupport is unique in its organization and function. It is the first organization in India that started the homecare visits for the terminal cancer patients. It has its headquarter with the administrative staff and a helpline that is officially active for about 8 hours a day for 5 days a week. The organization also has 10 homecare teams who are involved in the home visit. They have recently had an added support of community network officials. Each homecare team has a doctor, a nurse, and a counselor.

**Result:**

The total number of patients visited by the homecare teams of Cansupport in the year 2008-2009 was 1025. Total 104 patients were discharged. Out of 798 admissions last year, 384 patients were from IRCH (AIIMS). The helpline had 333 patients and others were just 81. Generally the team had to travel about 50-150 km a day. The number of visits range from four to seven per day. Generally the first visit of the team usually takes 90-120 min as the team takes time to understand the patient. The subsequent visits usually take 30-45 min. Usually, such patients stay with the team for a period of 1-2 months and then expire. Some patients stay with the team for 1-7 days.

**Conclusion:**

The eagerness of patients wanting the teams to reach their residence may be judged by the given figures. The total number of patients visited by the homecare teams of Cansupport in the year 2008-2009 was 1025. Out of them, there were about 104 patients who were discharged. The term discharge means that the patients were not interested in our visit or were not available in our subsequent visit. It has to be mentioned here that the service is a definite demand by society provided that the cost may be catered too.

## INTRODUCTION

Before the medieval period, there was hardly any palliative care done.[1] This was more so during the Hippocratic era. It was believed that if the doctors treated the dying person then he would not be able to keep a person alive. So doctors would not attend the dying. The Christians started off a very elementary type of palliative care. This idea is based on the command in the bible “As you did it to one of the least of these my brethren, you did it to me.”

In 1952, the Marie Curies Home sent doctors and nurses to the residence of patients for their care. The initial movement of modern hospice care has been done by Twycross in 1980 from The Royal Society of medicine.

The setting of palliative care may be detailed before going into the methodology of the exercise.

### Hospital

Hospitals are the dens of maintenance of the physical body. It lacks the emotional, social, and spiritual care of the individual. The hospital is the right place for the treatment of the physical body. But it may not be the right place for palliative care.

### Hospice

This is the place for the person to die comfortably. Here no active treatment is given to the person to get rid of the disease. Here the management is toward the pain or the symptom and trying to keep the person as comfortable as possible till his last days.

### Home-based palliative care

Expert advice is given at home. Here the patient is at home. The family members are counseled and trained for the final moments. The patient is managed so that he remains comfortable physically, socially, and spiritually. Here homecare teams reach the patient with various specialties to help the patient and family.

Home-based palliative care seems to be the management. Here the patient is the first priority of all work in the family. The patient has the feeling that the family is near and is also comforted by known surroundings. There are many psyches playing here such as known surroundings, known members or attendants, the comfort of home atmosphere; the last hours in the home may be a great relief.

This study has been done to see the effectiveness of homecare visits in terminal cancer patients. This involves many factors such as the patients' acceptability, the eagerness of the medical team to reach the home of the patient, the cost-effectiveness of the project, etc.

## MATERIALS AND METHODS

The medical team usually consists of a doctor, a nurse, and a councilor. The team usually tries to manage at all three levels-the clinical level with the help of the doctor, nursing with the help of the nurse, and the psychological turmoil of the family due to the presence of a dying patient by the counselor. This makes the medical team effective in sorting out the problems related to the dying patient. In the lower socioeconomic strata, the patients are helped by dispensing of medical aids in the form of medical equipment and medicines. After the patient expires, there is a bereavement visit, which is basically oriented to bring the family back to the normal stream of life.[2]

The other requirements in the project were a vehicle and a driver. Here it is important to mention that the organization has three vehicles of its own that have been donated to them. The fuel is being provided by Indian Oil Company. The payments of the drivers are being donated by various organizations. There are eight taxis. These taxis are on monthly hire basis. They usually come under the 2000 km range per month and their fuel is provided by the taxi owners. They are paid Rs. 16,000 per month. Generally a taxi travels around 800-1200 km a month.

## RESULTS

The eagerness of patients wanting the teams to reach their residence may be judged by the following figures. The total number of patients visited by the homecare teams of Cansupport in the year 2008-2009 was 1025 [Table 1]. Out of them, there were about 104 patients who were discharged. Here the term discharge means that the patients were not interested in our visit or were not available in our subsequent visit.

**Table 1:** Consolidated homecare statistics (2008-2009)

Month	Patient	Visits	Phone	Adm	IRCH adm	HLAd	OthAd	Death	Disc	Mor	Hp	Mp	Lp	D care
April	304	562	93	77	38	35	4	31	3	99	112	105	86	1
May	320	556	134	51	34	16	1	34	13	101	131	98	91	2
June	346	550	140	72	40	24	8	39	7	115	118	133	95	0
July	361	653	180	64	44	13	7	55	14	116	124	130	107	0
Aug	359	676	151	64	32	22	10	32	13	111	120	154	85	2
Sept	383	739	152	70	27	37	6	48	13	120	127	143	113	4
Oct	385	715	159	57	32	22	3	42	2	108	136	153	96	4
Nov	402	655	155	56	24	24	8	51	6	113	167	141	94	2
Dec	435	749	157	90	45	38	7	58	3	119	166	182	87	0
Jan	456	638	146	71	30	33	8	44	15	122	185	173	98	1
Feb	461	531	101	59	21	32	6	33	13	122	164	179	86	0
Mar	475	698	106	67	17	37	13	40	2	127	162	189	88	1
Total	1025	7722	1674	798	384	333	81	507	104	1373	-	-	-	17

Adm = Admission; IRCH Adm = IRCH admissions; HLAd = Helpline admission; OthAd = Other admission; Disc = Discharge; Mor = Morphine; Hp = High priority; Mp = Medium priority; Lp = Low priority; D care = Day care

Those who were not interested in our visits were patients either having social problems such as their families were dysfunctional or the patients were being badly treated by the relatives. These patients just catered to around 20-25% of the total discharge.

The remaining discharges were in the category of being not available. Here most of the patients were terminal and went back to their village for better quality of life in their final days.

The cost of a single visit to the patient would be approximately Rs. 500/-.

Generally the team has to travel about 50-150 km a day. The number of visits range from four to seven per day. Usually the first visit of the team usually takes 90-120 min as the team takes time to understand the patient. The subsequent visits usually take 30-45 min.

**Graph 1:**
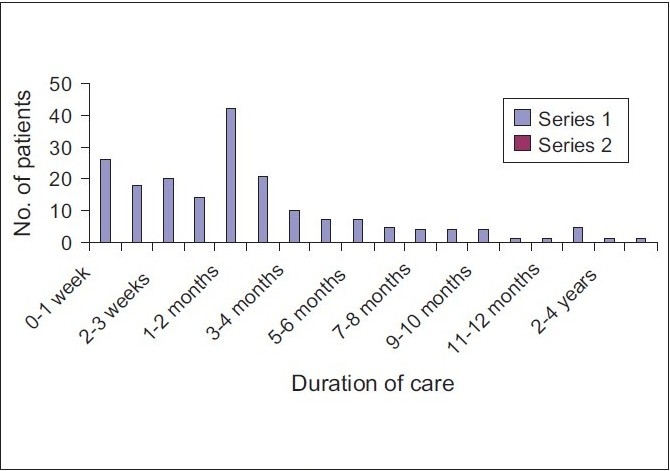
Life expectancy graph

Usually the patients stay with the team for a period of 1-2 months and then expire. Some patients stay with the team for 1-7 days. The patients in the second category are in their terminal phase and the family members are apprehensive how the end would be for the patient. That is why the teams are called in at this point of time in the patients' life [Graph 1].

## DISCUSSION

The cost-effectiveness of the project might not be measured in terms of money. One has to realize that there are many lacunas in the medical system of the nation. This is one of the ways to overcome one of the most important lacunas, the aid to the poorest sections of society in an effective manner. This is the place where medical aid is needed the most. We must remember here that a visit costs about Rs. 500/- to the organization.[3]

Home-based palliative care seems to be the ultimate stage of palliative care management theoretically. It is definitely the most advanced care compared to hospice and hospital management. Comparing it with self-contained medical skills of society (eg., Kerala medical system of society), it is definitely more advanced as expert advice is reaching the patients at their door step.

The importance of palliative care lies in understanding the psychological, medical, and social aspects of the patient and the family. The homecare teams are fully equipped to cater to the above requirements. If the patient is uncomfortable, then the whole family is in turmoil. This might be physical or psychological. The physical discomfort is usually taken care by the doctor and the nurse. Once the patient becomes comfortable physically, then only he can start thinking.

Once the physical comfort is taken care off, the patient would like to know whether he would live or not. Some patients are kept in the dark about the span of life. The family members are usually apprehensive about the decision of letting the patient know about his lifespan. It is important to convince the family members that it is always advisable to let the patient know about the life expectancy. There are many reasons to it. The patient might want last wishes to be made, which the family members may be able to cater to.

The availability of the homecare teams to society has the distinct advantage of feeling the pulse of society. The homecare team is able to judge the palliative requirement of the society. In fact, it may be able to give an idea of many other features of the particular society. Being a specialized team, it might be able to predict the disease state of the society.[4]

The drawback of the homecare team is that it is not able to cater to emergencies. The calls are not an immediate response to the patients' need. They would not be able to make a visit the day the call is made. The homecare team does have a mobile that the doctor is able to respond if he has seen the patient. The system needs a considerable amount of investment, skill, and energy.[5]

## CONCLUSION

The year 2008-2009 has been a landmark year for Cansupport where there has been an increase in the number of teams from five to ten. In spite of the increase in the number of teams, they are having difficulty in making one visit per week for a single patient wherever the need is, as the number of the patients are increasing. Here one has to remember that the majority of the patients are still from AIIMS. Out of 798 admissions last year, 384 were from IRCH (AIIMS). The helpline had 333 and others were just 81. The other hospitals have still not been informed as yet in toto. So if done entirely, definitely there would be a greater need of teams and skilled manpower.
